# Childhood adversities and post-traumatic stress: predictive pathways through acute stress disorder

**DOI:** 10.1192/bjo.2025.10948

**Published:** 2026-01-16

**Authors:** Josleen Al Barathie, Majd Chamoun, Rayane Osman, Elie G. Karam

**Affiliations:** Institute for Development, Research, Advocacy and Applied Care (IDRAAC), Beirut, Lebanon; Department of Psychiatry and Clinical Psychology, Saint George Hospital University Medical Center, Beirut, Lebanon; Department of Psychiatry and Clinical Psychology, Saint George University of Beirut, Faculty of Medicine, Beirut, Lebanon

**Keywords:** Acute stress disorder, post-traumatic stress disorder, subthreshold post-traumatic stress disorder, childhood adversities, Beirut port blast

## Abstract

**Background:**

Trauma, a psychological phenomenon, can contribute to several mental health disorders such as acute stress disorder (ASD) and post-traumatic stress disorder (PTSD). Understanding the impacts of childhood adversities on mental disorders is essential, since early trauma can profoundly shape an individual’s reaction to subsequent trauma.

**Aims:**

To study the association between childhood adversities and full-threshold and subthreshold PTSD onset, either directly or through the intermediary role of ASD.

**Method:**

Data were collected longitudinally from healthcare workers aged 18 years and above, working at Saint George Hospital University Medical Center, at three distinct time points: 9–15 days (wave 1), 21–27 days (wave 2) and 6–7 months (wave 3) following the Beirut port blast on 4 August 2020.

Childhood adversities, encompassing physical, emotional and sexual abuse, were collected. ASD and PTSD were assessed, respectively, using the Acute Stress Disorder Scale and the PTSD Checklist for DSM-5. Odds ratios, both unadjusted and adjusted, with 95% confidence intervals, were calculated to study the associations.

**Results:**

Emotional abuse was found to be a significant predictor of ASD at wave 2 (21–27 days), and both full-threshold and subthreshold PTSD at wave 3 (6–7 months) post-blast. Individuals with negative ASD status at waves 1 and 2 who experienced emotional abuse in childhood showed approximately double the rates of subthreshold PTSD.

**Conclusions:**

Childhood emotional abuse was a strong predictor of both ASD and PTSD. Screening for past childhood adversities is important, even in individuals without ASD, as they remain more vulnerable to later PTSD.

Trauma, in contemporary psychiatric nosology, is defined as exposure to ‘actual or threatened death, serious injury, or sexual violence’, whether directly experienced, witnessed or learned about, and is often followed by persistent intrusion symptoms, avoidance, negative mood or cognition, and heightened arousal.^1^ Although traumatic events can occur in any population, certain groups, including survivors of armed conflict, disaster victims, first responders and healthcare workers, are disproportionately exposed.^2^ This makes them more susceptible to developing trauma-related disorders. From those groups, healthcare workers in particular face a unique constellation of risk factors: repeated encounters with suffering, death and critical incidents; high-stakes decision-making under time pressure; and frequent exposure to workplace violence or verbal aggression.^3^ Furthermore, the stigma surrounding mental health in the healthcare profession often discourages workers from seeking help, as they may fear judgement, loss of credibility or professional repercussions, thereby exacerbating the risk of chronic psychological distress.^4^

## Responses to trauma

Individuals exhibit a broad spectrum of reactions to trauma, ranging from negative to neutral, and even potentially positive ones. Following a traumatic event, individuals may experience fear and anxiety, emotional numbness, anger, guilt and feelings of losing control.^5^ For some, these reactions diminish naturally, without progressing into a psychiatric disorder. However, others may experience a more severe and persistent impact, including ongoing anxiety, anhedonia, dysphoria, externalisations of anger, aggressiveness and dissociation, which can ultimately lead to the development of a disorder.^6^

A range of psychiatric disorders can follow trauma, with some being more common than others. Post-traumatic stress disorder (PTSD) and acute stress disorder (ASD) occur frequently and may coexist with other disorders such depression, anxiety and substance misuse.^7^ Alternatively, some people experience post-traumatic growth,^8^ with some studies suggesting a ‘toughening effect’ of prior stressors that enhances resilience and desensitisation to future trauma.^9,10^

## Factors influencing trauma reactions

The factors influencing these disparate reactions are multifaceted. The Conceptual Framework for the Impact of Traumatic Experiences^11^ identifies five key elements shaping trauma response: individual biological factors, developmental stage during trauma, trauma severity, social context before and after trauma, and life events preceding and following trauma. Regarding prior life events, the model suggests that earlier stressful experiences may either moderate or sensitise responses to future trauma, depending on the perceived controllability and negativity of the trauma. Previous experiences that make a traumatic event appear more manageable have a moderating effect, whereas those that make it seem less manageable have a sensitising effect.^11^

Moreover, the timing of these events is crucial, particularly when they occur during childhood. Children are particularly vulnerable because of to their limited coping mechanisms and ability to express complex emotions.^12^ The dynamic brain development during childhood, especially during the preschool years and adolescence, makes this period especially sensitive to trauma’s effects.^13^ Alteration in neural development resulting from early trauma can disrupt cognitive and emotional regulation processes essential for adaptive functioning later in life.^14^

## Adverse childhood experiences

Stressful life events during childhood are known as adverse childhood experiences (ACEs). They occur before the age of 18 years and involve eight domains: physical, sexual and emotional abuse; having a depressed, mentally ill or suicidal household member; alcohol or drug misuse within the household; a family member being incarcerated; violence between adults in the household and parental divorce or separation.^15^

Understanding the impact of childhood adversities is crucial for comprehensively addressing their long-term impact on mental health outcomes, particularly the development of later ASD and PTSD.

## Link between childhood adversities and PTSD

Childhood adversities are strongly linked to an increased risk of PTSD. McLaughlin et al^16^ analysed data from 27 017 participants in the World Mental Health Surveys and found that four specific childhood adversities (physical and sexual abuse, neglect, parent psychopathology) were associated with a 1.8 times higher likelihood of PTSD following traumatic experiences. Moreover, numerous studies have emphasised that individuals with a history of childhood trauma are more susceptible to develop PTSD after exposure to subsequent distressing events.^17,18^

## Link between childhood adversities and ASD

Although research on the relationship between childhood adversities and the risk of ASD is still limited, emerging evidence indicates that individuals who experienced trauma during childhood are at a higher risk of developing ASD following trauma in adulthood. A retrospective cohort study conducted by Wang et al found that individuals with a history of childhood abuse were 3.27 times more likely to develop ASD/PTSD later in life.^19^ Another study by Maunder et al measured acute stress response over a 2-week period following critical incidents during paramedic duties. This study found that those with a history of childhood abuse/neglect exhibited heightened stress responses to such events.^20^

## Aims

Building on previous literature that predominantly examined childhood adversity in the prediction of full threshold DSM-5 PTSD and, to a much lesser extent, ASD, this research offers novel insights on the longitudinal investigation of the impact of childhood adversities (particularly physical abuse, sexual abuse and neglect) on future PTSD onset, both with and without preceding ASD. Secondarily, this study did not only examine full threshold DSM-5 PTSD, but also subthreshold PTSD, which is gaining wider attention, alongside the assessment of ASD at two distinct time intervals.

## Method

### Participants

The Beirut port blast, which occurred on 4 August 2020, was estimated to be one of the strongest non-nuclear explosions ever recorded, resulting in hundreds of deaths and thousands of injuries. One of the most extensively damaged healthcare centres was Saint George Hospital University Medical Center, in Beirut Lebanon (SGHUMC). The participants in this study comprised healthcare workers aged 18 years and above, employed at SGHUMC, including clinicians, administrative staff and supportive personnel. The participants are individuals who witnessed first hand the aftermath of the blast and encountered scenes of devastation such as casualties, mutilation, destruction and widespread chaos.

### Procedure

The study encompassed three waves of data collection, initiated in the aftermath of the Beirut port explosion. The first two waves were part of a national initiative aimed at assessing the impact of the explosion on the acute stress of the healthcare workers. During wave 1 and following an email communication from SGHUMC, all staff members were requested to report for COVID-19 testing 9–15 days post-blast, the data were gathered through face-to-face interactions after witnessing and formally recording the verbal consent. Given the urgency of the situation in wave 1 and the need to minimise burden on participants experiencing significant physical and psychological stress, we opted for verbal consent as a fast and feasible approach. This method allowed participants to provide informed agreement without being overwhelmed by lengthy procedures, ensuring both ethical and practical considerations were respected. Wave 2 occurred between 21 and 27 days post-blast, with data collection conducted via an online survey following a signed consent. Similarly, wave 3, which transpired 6–7 months post-blast, involved data collection through an online survey following a signed consent. In wave 3 specifically, proxy PTSD secondary to the explosion was assessed, alongside previous trauma experiences, such as childhood adversities encompassing emotional, sexual and physical trauma. Notably, the third wave was an international effort as part of the Health Care Workers cohort study. Further information is present elsewhere.^21^

We chose the time points of 9–15 days (wave 1), 21–27 days (wave 2) and 6–7 months (wave 3) following the Beirut port blast based on clinical definitions and the natural progression of stress-related disorders. ASD is characterised by symptoms occurring within the first month after trauma, which justifies the early waves at 9–15 and 21–27 days. In contrast, proxy PTSD is defined by symptoms that persist beyond a month, which is why we included the 6–7 month follow-up (wave 3) to capture more chronic symptoms.

### Measures

The questionnaire utilised in this study encompassed sociodemographic information (age and gender), childhood adversities and mental health measures for ASD and proxy PTSD.

The childhood adversities included parental physical abuse, parental neglect/emotional abuse and childhood sexual abuse. It is to be noted that neglect is recognised as part of the emotional abuse domain of childhood maltreatment rather than as a distinct category, given their overlapping definitions, shared mechanisms and frequent combined use in research as ‘emotional abuse/neglect’.^22^ In Glaser’s conceptual framework for identifying emotional maltreatment, emotional abuse and neglect are jointly conceptualised under psychological maltreatment, encompassing both acts of commission (emotional abuse) and omission (emotional neglect).^23^

In the first month following the blast, ASD was evaluated with the Acute Stress Disorder Scale (ASDS), a self-report measurement with established psychometric characteristics.^24^ The ASDS consists of 21 items, 19 of which assess symptoms of acute stress following a trauma. Each item is rated on a five-point scale ranging from 1 (not at all) to 5 (very much). Participants were asked to indicate the extent to which they experienced symptoms such as intrusion, negative mood, dissociation, avoidance and arousal, with higher scores indicating greater severity. To derive a probable DSM-5 diagnosis of ASD, participants were required to endorse at least nine out of 14 relevant symptoms. The ASDS was translated into Arabic by the research team, and demonstrated excellent internal consistency.

Between 6 and 7 months after the blast, proxy PTSD was assessed with the PTSD Checklist for DSM-5 (PCL-5).^25^ This 20-item self-report measure evaluates the 20 DSM-5 symptoms of proxy PTSD, with each symptom rated on a scale of 0 to 4, corresponding to various levels of severity. The diagnostic criteria for proxy PTSD were based on the endorsement of symptoms across different clusters (B–E), with specific thresholds required for each cluster. Additionally, two definitions of subthreshold PTSD were employed: the ‘six plus’ definition, requiring at least six symptoms, and the ‘majority’ definition, necessitating meeting full threshold for three out of four criteria.^26^

We would like to note that although the PCL-5 closely aligns with the DSM-5 diagnostic criteria for PTSD, as its items directly map onto the symptom clusters defined in the manual, it is still a screening instrument rather than a diagnostic tool, which is why we use the term *proxy diagnosis* throughout the manuscript, to reflect that PTSD was inferred based on PCL-5 rather than confirmed through a structured clinical interview/diagnosis.

### Analysis

Data analysis was conducted to examine the predictive relationship between childhood adversities and ASD at 9–15 days and 2127 days following the blast, as well as proxy PTSD at 6–7 months after the blast. Initially, each childhood adversity was examined individually, categorised as ‘yes’ or ‘no’. Subsequently, the childhood adversities were combined into a single variable indicating the presence or absence of any childhood adversity.

### Model specification

#### Model 1

The first model employed a bivariate approach, assessing the predictive capacity of each individual childhood adversity.

#### Model 2

The second model incorporated all three childhood adversities simultaneously to evaluate their combined predictive power.

#### Model 3

In the third model, age and gender were added as covariates to control for potential confounding.

Following the initial analysis, a similar approach was applied, but instead of examining each childhood adversity separately, the analysis was conducted using the presence of any childhood adversity as the predictor variable. This process was repeated across various models, accounting for age and gender.

Furthermore, the analysis was stratified into two time frames: ASD status 9–15 days post-blast (wave 1) and 21–27 days post-blast (wave 2) to explore potential differences in the predictive relationship between childhood adversities and proxy PTSD (wave 3).

Odds ratios, *P*-values and 95% confidence intervals were calculated to quantify the strength and significance of the associations. A *P*-value <0.05 was considered statistically significant. Missing data were handled using complete-case analysis. All statistical analyses were conducted with Stata version 17 for Windows (StataCorp, College Station, Texas, USA; https://www.stata.com).

### Ethics

The authors assert that all procedures contributing to this work comply with the ethical standards of the relevant national and institutional committees on human experimentation and with the Helsinki Declaration of 1975, as revised in 2013. All procedures involving human patients were approved by the Institutional Review Board committee of the SGHUMC Faculty of Medicine, University of Balamand, Lebanon, which is registered with the US Office of Human Research Protections in the Department of Health and Human Services.

## Results

### Descriptive

The questionnaire was completed by 570, 733 and 808 individuals 9–15 days, 21–27 days and 6–7 months after the blast, respectively. At 21–27 days and 6–7 months, the total number of workers at the hospital was 1927, corresponding to a response rate of 38 and 41.88%, respectively. These numbers represent the total participants assessed at each wave of this open longitudinal study, in which new participants could be recruited at later time points rather than restricting the sample to a fixed cohort. The present analysis focuses on the 426 participants who were followed up across all three time frames. In wave 3, out of the total number of 808 respondents, we have information on 426 participants (50 exclusively followed up from wave 1; 209 exclusively followed up from wave 2 and 167 from waves 1 and 2). For further details, see Supplementary Table 1 available at https://doi.org/10.1192/bjo.2025.10948.

The data showed a mean age of approximately 36.90±12.36 years, and the majority were female (70.72%). Childhood adversities were reported by nearly a fifth of the participants: 22.21% experienced parental physical abuse (‘hit by parents’), 21.18% reporting emotional abuse (‘neglected’) and 9.41% reported sexual abuse (‘sexually abused’). The prevalence of ASD, assessed with the ASDS, was higher in wave 1 at 9–15 days post-blast (38.34%) compared with wave 2, which was 21–27 days post-blast (30%). At 6–7 months post-blast, 27.35% met the criteria for full threshold DSM-5 proxy PTSD based on the PCL-5, whereas 48.33% met the ‘majority’ subthreshold criteria and 51.94% met the ‘six plus’ subthreshold criteria.

### Using childhood adversity as a separate predictor

#### Childhood adversity as a predictor of ASD 9–15 days post-blast

When studied individually, sexual abuse was the only childhood adversity that approached statistical significance as a predictor of ASD at 9–15 days post blast when accounting for all three childhood adversities in the model (model 2), with an odds ratio of 2.59 (*P* = 0.058, 95% CI 0.46–0.96) (Table 1). After adjusting for potential confounders, the odds ratio remained twice as high, yet, it was no longer significant (*P* = 0.153, 95% CI 0.76–5.83).

**Table 1:** Logistic regression models: prediction of ASD (wave 1), ASD (wave 2) and full-threshold and subthreshold PTSD (wave 3), using childhood adversities as separate entities

Independent variables	Model 1^**^			Model 2^***^			Model 3^****^		
	Odds ratio	*P*-value	95% CI	Odds ratio	*P*-value	95% CI	Odds ratio	*P*-value	95% CI
**Dependent variable: ASD at wave 1**									
Hit by parents									
Yes	1.24	0.547	(0.61, 2.51)	0.56	0.251	(0.21, 1.51)	0.55	0.303	(0.21, 1.62)
No (reference)	–	–	–	–	–	–	–	–	–
Neglected									
Yes	1.97	0.065	(0.96, 4.03)	2.36	0.079	(0.90, 6.17)	2.34	0.090	(0.87, 6.31)
No (reference)	–	–	–	–	–	–	–	–	–
Sexually abused									
Yes	2.64	0.038^*^	(1.05, 6.62)	2.59	0.058^*^	(0.46, 0.96)	2.10	0.153	(0.76, 5.83)
No (reference)	–	–	–	–	–	–	–	–	–
Age	0.97	0.050^*^	(0.95, 1.00)	–	–	–	0.97	0.034^*^	(0.94, 0.99)
Gender									
Male	0.39	0.021^*^	(0.18, 0.87)	–	–	–	0.39	0.045^*^	(0.16, 0.98)
Female (reference)	–	–	–				–	–	–
**Dependent variable: ASD at wave 2**									
Hit by parents									
Yes	1.20	0.532	(0.68, 2.10)	0.48	0.071	(0.22, 1.06)	0.45	0.063	(0.19, 1.04)
No (reference)	–	–	–	–	–	–	–	–	–
Neglected									
Yes	2.63	0.001^*^	(1.52, 4.53)	3.96	0.000^*^	(1.91, 8.22)	3.93	0.001^*^	(1.81, 8.54)
No (reference)	–	–	–	–	–	–	–	–	–
Sexually abused									
Yes	1.42	0.401	(0.63, 3.23)	1.17	0.738	(0.47, 2.86)	1.15	0.773	(0.45, 2.89)
No (reference)	–	–	–	–	–	–	–	–	–
Age	0.97	0.001^*^	(0.95, 0.99)	–	–	–	0.96	0.001^*^	(0.93, 0.98)
Gender									
Male	0.53	0.029^*^	(0.20, 0.94)	–	–	–	0.48	0.037^*^	(0.24, 0.96)
Female (reference)	–	–	–				–	–	–
**Dependent variable: PTSD full**									
Hit by parents									
Yes	1.21	0.501	(0.69, 2.13)	0.65	0.263	(0.300, 1.39)	0.68	0.337	(0.31, 1.50)
No (reference)	–	–	–	–	–	–	–	–	–
Neglected									
Yes	2.13	0.006^*^	(1.24, 3.68)	2.87	0.004^*^	(1.40, 5.86)	2.60	0.011^*^	(1.24, 5.43)
No (reference)	–	–	–	–	–	–	–	–	–
Sexually abused									
Yes	1.10	0.819	(0.49, 2.44)	0.88	0.774	(0.37, 2.10)	0.90	0.824	(0.37, 2.19)
No (reference)	–	–	–	–	–	–	–	–	–
Age	1.00	0.661	(0.99, 1.02)	–	–	–	1.00	0.940	(0.98, 1.02)
Gender									
Male	0.55	0.042^*^	(0.31, 0.98)	–	–	–	0.63	0.167	(0.33, 1.21)
Female (reference)	–	–	–				–	–	–
**Dependent variable: PTSD majority**									
Hit by parents									
Yes	1.70	0.035^*^	(1.04, 2.10)	0.84	0.607	(0.44, 1.63)	0.84	0.611	(0.43, 1.65)
No (reference)	–	–	–	–	–	–	–	–	–
Neglected									
Yes	2.71	0.000^*^	(1.62, 4.53)	2.76	0.002^*^	(1.44, 5.30)	2.41	0.009^*^	(1.24, 4.69)
No (reference)	–	–	–	–	–	–	–	–	–
Sexually abused									
Yes	2.11	0.036^*^	(1.05, 4.25)	1.56	0.254	(0.73, 3.33)	1.51	0.298	(0.69, 3.28)
No (reference)	–	–	–	–	–	–	–	–	–
Age	0.99	0.326	(0.97, 1.01)	–	–	–	0.99	0.353	(0.98, 1.01)
Gender									
Male	0.61	0.044^*^	(0.37, 0.99)	–	–	–	0.67	0.145	(0.39, 1.15)
Female (reference)	–	–	–				–	–	–
**Dependent variable: PTSD six symptoms**									
Hit by parents									
Yes	1.69	0.038^*^	(1.03, 2.77)	0.94	0.843	(0.49, 1.79)	0.98	0.942	(0.50, 1.89)
No (reference)	–	–	–	–	–	–	–	–	–
Neglected									
Yes	2.53	0.000^*^	(1.51, 4.24)	2.54	0.005^*^	(1.33, 4.86)	2.36	0.011^*^	(1.21, 4.56)
No (reference)	–	–	–	–	–	–	–	–	–
Sexually abused									
Yes	1.66	0.153	(0.83, 3.32)	1.19	0.655	(0.56, 2.53)	1.31	0.491	(0.60, 2.85)
No (reference)	–	–	–	–	–	–	–	–	–
Age	1.0	0.903	(0.98, 1.02)	–	–	–	1.00	0.914	(0.98, 1.02)
Gender									
Male	0.60	0.038^*^	(0.37, 0.97)	–	–	–	0.68	0.162	(0.40, 1.16)
Female (reference)	–	–	–				–	–	–

ASD, acute stress disorder; PTSD, post-traumatic stress disorder.

* Significant at <0.05.

** Bivariable logistic regression model of the dependent variable on each of the independent variables.

*** Multivariable logistic regression model of the dependent variable on hit by parents, neglected and sexually abused.

**** Multivariable logistic regression model of the dependent variable on hit by parents, neglected, sexually abused, age and gender.

#### Childhood adversity as a predictor of ASD 21–27 days post-blast

At 21–27 days post-blast, emotional abuse was a significant predictor of ASD, and this was retained after adjusting for confounders and other childhood adversities (model 3) (odds ratio 3.93, *P* = 0.001, 95% CI 1.81–8.54) (Table 1).

#### Childhood adversity as a predictor of PTSD 6-7 months post-blast

At 6–7 months, emotional abuse was found to be a significant predictor of full threshold DSM-5 proxy PTSD (odds ratio 2.60, *P* = 0.011, 95% CI 1.24–5.43), of subthreshold ‘majority’ PTSD (odds ratio 2.41, *P* = 0.009, 95% CI 1.24–4.69) and of subthreshold ‘six plus’ PTSD (odds ratio 2.36, *P* = 0.011, 95% CI 1.21–4.56) (Table 1). These associations persisted after adjusting for potential confounders and other adversities.

### Using childhood adversity as a binary variable

#### Total childhood adversities as a predictor of proxy PTSD 6–7 months post-blast

When any childhood adversity (versus none) was used as the exposure, it significantly predicted subthreshold proxy PTSD at 6–7 months. Specifically, the odds ratios were 1.66 (*P* = 0.003, 95% CI 1.19–2.32) for ‘majority’ proxy PTSD and 1.55 (*P* = 0.011, 95% CI 1.11–2.17) for ‘six plus’ proxy PTSD after controlling for age and gender (Table 2).

**Table 2:** Logistic regression models: prediction of ASD (wave 1), ASD (wave 2) and full-threshold and subthreshold PTSD (wave 3), using childhood adversities as a binary variable

Independent variables	Model 1^**^			Model 3^***^		
	Odds ratio	*P*-value	95% CI	Odds ratio	*P*-value	95% CI
**Dependent variable: ASD at wave 1**						
Childhood adversities						
At least one type	1.54	0.179	(0.82, 2.91)	1.44	0.278	(0.74, 2.80)
No (reference)	–	–	–	–	–	–
Age	0.97	0.050^*^	(0.95, 1.00)	0.97	0.035^*^	(0.94, 1.00)
Gender						
Male	0.39	0.021^*^	(0.18, 0.87)	0.37	0.030^*^	(0.15, 0.90)
Female (reference)	–	–	–			
**Dependent variable: ASD at wave 2**						
Childhood adversities						
At least one type	1.58	0.073	(0.96, 2.62)	1.48	0.154	(0.86, 2.52)
No (reference)	–	–	–	–	–	–
Age	0.97	0.001^*^	(0.95, 0.99)	0.96	0.002^*^	(0.94, 0.99)
Gender						
Male	0.53	0.029^*^	(0.20, 0.94)	0.47	0.028^*^	(0.24, 0.92)
Female (reference)	–	–	–	–	–	–
**Dependent variable: PTSD full**						
Childhood adversities						
At least one type	1.37	0.080	(0.96, 1.95)	1.40	0.071	(0.97, 2.01)
No (reference)	–	–	–	–	–	–
Age	1.00	0.661	(0.99, 1.02)	1.00	0.695	(0.99, 1.02)
Gender						
Male	0.55	0.042^*^	(0.31, 0.98)	0.60	0.016^*^	(0.40, 0.91)
Female (reference)	–	–	–	–	–	–
**Dependent variable: PTSD majority**						
Childhood adversities						
At least one type	1.68	0.002^*^	(1.22, 2.33)	1.66	0.003^*^	(1.19, 2.32)
No (reference)	–	–	–	–	–	–
Age	0.99	0.326	(0.97, 1.01)	0.99	0.373	(0.98, 1.01)
Gender						
Male	0.61	0.044^*^	(0.37, 0.99)	0.55	0.001^*^	(0.39, 0.79)
Female (reference)	–	–	–	–	–	–
**Dependent variable: PTSD six symptoms**						
Childhood adversities						
At least one type	1.51	0.013^*^	(1.09, 2.08)	1.55	0.011^*^	(1.11, 2.17)
No (reference)	–	–	–	–	–	–
Age	1.0	0.903	(0.98, 1.02)	0.99	0.437	(0.98, 1.01)
Gender						
Male	0.60	0.038^*^	(0.37, 0.97)	0.53	0.000^*^	(0.38, 0.75)
Female (reference)	–	–	–			

ASD, acute stress disorder; PTSD, post-traumatic stress disorder.

* Significant at *P*<0.05.

** Bivariable logistic regression model of the dependent variable on each of the independent variables.

*** Multivariable logistic regression model of the dependent variable on childhood adversities, age and gender.

#### Childhood adversity as a predictor of proxy PTSD: stratification by ASD wave 1 and ASD wave 2

Analyses stratified by ASD status at waves 1 and 2 showed that among participants with ASD at both 9–15 and 21–27 days, having any childhood adversity did not predict either full-threshold or subthreshold proxy PTSD.

However, among individuals with negative ASD at 9–15 days post-blast, having at least one childhood adversity predicted subthreshold proxy PTSD (majority: odds ratio 3.02, *P* = 0.04, 95% CI 1.05–8.69) (Table 3), after controlling for age and gender.

**Table 3:** Logistic regression models: prediction of full-threshold and subthreshold PTSD by childhood adversities as a binary variable: stratified by ASD at wave 1

Independent variables	ASD at wave 1 negative						ASD at wave 1 positive					
	Model 1^**^			Model 3^****^			Model 1^**^			Model 3^****^		
	Odds ratio	*P*-value	95% CI	Odds ratio	*P*-value	95% CI	Odds ratio	*P*-value	95% CI	Odds ratio	*P*-value	95% CI
**Dependent variable: PTSD full**												
Childhood adversities												
At least one type	1.38	0.671	(0.31, 6.23)	1.56	0.574	(0.33, 7.37)	0.70	0.484	(0.25, 1.92)	0.77	0.630	(0.27, 2.21)
No (reference)	–	–	–	–	–	–	–	–	–	–	–	–
Age	1.01	0.650	(0.95, 1.07)	1.02	0.576	(0.96, 1.07)	1.02	0.264	(0.98, 1.07)	1.03	0.201	(0.98, 1.08)
Gender												
Male	1.67	0.490	(0.39, 7.22)	2.38	0.279	(0.50, 11.40)	0.53	0.440	(0.10, 2.67)	0.94	0.949	(0.17, 5.38)
Female (reference)	–	–	–	–	–	–	–	–	–	–	–	–
**Dependent variable: PTSD majority**												
Childhood adversities												
At least one type	3.00	0.030^*^	(1.11, 8.09)	3.02	0.040^*^	(1.05, 8.69)	1.21	0.684	(0.48, 3.05)	1.36	0.529	(0.52, 3.59)
No (reference)	–	–	–	–	–	–	–	–	–	–	–	–
Age	1.02	0.396	(0.98, 1.06)	1.02	0.326	(0.98, 1.07)	1.01	0.514	(0.97, 1.06)	1.02	0.375	(0.97, 1.07)
Gender												
Male	0.85	0.772	(0.28, 2.60)	1.43	0.562	(0.43, 4.81)	0.45	0.297	(0.10, 2.02)	0.49	0.377	(0.10, 2.36)
Female (reference)	–	–	–	–	–	–	–	–	–	–	–	–
**Dependent variable: PTSD six symptoms**												
Childhood adversities												
At least one type	2.21	0.128	(0.80, 6.12)	2.39	0.121	(0.79, 7.23)	0.84	0.716	(0.33, 2.13)	1.08	0.881	(0.40, 2.88)
No (reference)	–	–	–	–	–	–	–	–	–	–	–	–
Age	1.01	0.576	(0.97, 1.06)	1.02	0.472	(0.97, 1.06)	1.04	0.117	(0.99, 1.09)	1.05	0.098	(0.99, 1.10)
Gender												
Male	0.69	0.545	(0.21, 2.92)	1.22	0.766	(0.34, 4.40)	0.57	0.453	(0.13, 2.46)	0.77	0.739	(0.17, 3.54)
Female (reference)	–	–	–	–	–	–	–	–	–	–	–	–

PTSD, post-traumatic stress disorder; ASD, acute stress disorder.

* Significant at <0.05.

** Bivariable logistic regression model of the dependent variable on each of the independent variables.

**** Multivariable logistic regression model of the dependent variable on childhood adversities, age and gender.

Similarly, among individuals with negative ASD at 21–27 days post-blast, having at least one childhood adversity predicted subthreshold proxy PTSD (majority: odds ratio 2.65, *P* = 0.003, 95% CI 1.41–4.99; six plus: odds ratio 1.89, *P* = 0.048, 95% CI 1.00–3.56), after controlling for confounders (Table 4).

**Table 4:** Logistic regression models: prediction of full-threshold and subthreshold PTSD by childhood adversities as a binary variable: stratified by ASD at wave 2

Independent variables	ASD at wave 2 negative						ASD at wave 2 positive					
	Model 1^**^			Model 3^***^			Model 1^**^			Model 3^***^		
	Odds ratio	*P*-value	95% CI	Odds ratio	*P*-value	95% CI	Odds ratio	*P*-value	95% CI	Odds ratio	*P*-value	95% CI
**Dependent variable: PTSD full**												
Childhood adversities												
At least one type	1.98	0.090	(0.90, 4.35)	2.23	0.056^*^	(0.98, 5.07)	0.90	0.802	(0.39, 2.05)	0.78	0.576	(0.33, 1.86)
No (reference)	–	–	–	–	–	–	–	–	–	–	–	–
Age	1.04	0.004^*^	(1.01, 1.07)	1.04	0.029^*^	(1.00, 1.07)	0.98	0.338	(0.94, 1.02)	1.00	0.943	(0.96, 1.04)
Gender												
Male	0.54	0.167	(0.23, 1.29)	0.39	0.104	(0.13, 1.21)	0.79	0.643	(0.29, 2.14)	1.58	0.444	(0.49, 5.10)
Female (reference)	–	–	–	–	–	–	–	–	–	–	–	–
**Dependent variable: PTSD majority**												
Childhood adversities												
At least one type	2.73	0.001^*^	(1.48, 5.03)	2.65	0.003^*^	(1.41, 4.99)	2.45	0.091	(0.87, 6.91)	2.11	0.172	(0.73, 6.16)
No (reference)	–	–	–	–	–	–	–	–	–	–	–	–
Age	1.01	0.308	(0.99, 1.03)	1.01	0.320	(0.99, 1.04)	0.99	0.514	(0.94, 1.03)	0.99	0.553	(0.94, 1.03)
Gender												
Male	0.69	0.248	(0.36, 1.30)	0.63	0.211	(0.31, 1.30)	1.42	0.607	(0.37, 5.46)	2.22	0.332	(0.44, 11.08)
Female (reference)	–	–	–	–	–	–	–	–	–	–	–	–
**Dependent variable: PTSD six symptoms**												
Childhood adversities												
At least one type	1.73	0.076	(0.94, 3.18)	1.89	0.048^*^	(1.00, 3.56)	2.79	0.068	(0.93, 8.39)	2.54	0.107	(0.82, 7.86)
No (reference)	–	–	–	–	–	–	–	–	–	–	–	–
Age	1.02	0.088	(1.0, 1.04)	1.02	0.147	(1.00, 1.04)	1.0	0.959	(0.95, 1.05)	1.00	0.994	(0.95, 1.05)
Gender												
Male	0.65	0.189	(0.35, 1.23)	0.63	0.198	(0.31, 1.28)	2.16	0.333	(0.45, 10.29)	5.23	0.12	(0.62, 43.59)
Female (reference)	–	–	–	–	–	–	–	–	–	–	–	–

PTSD, post-traumatic stress disorder; ASD, acute stress disorder.

* Significant at *P*<0.05.

** Bivariable logistic regression model of the dependent variable on each of the independent variables.

*** Multivariable logistic regression model of the dependent variable on childhood adversities, age and gender.

**Fig. 1:**
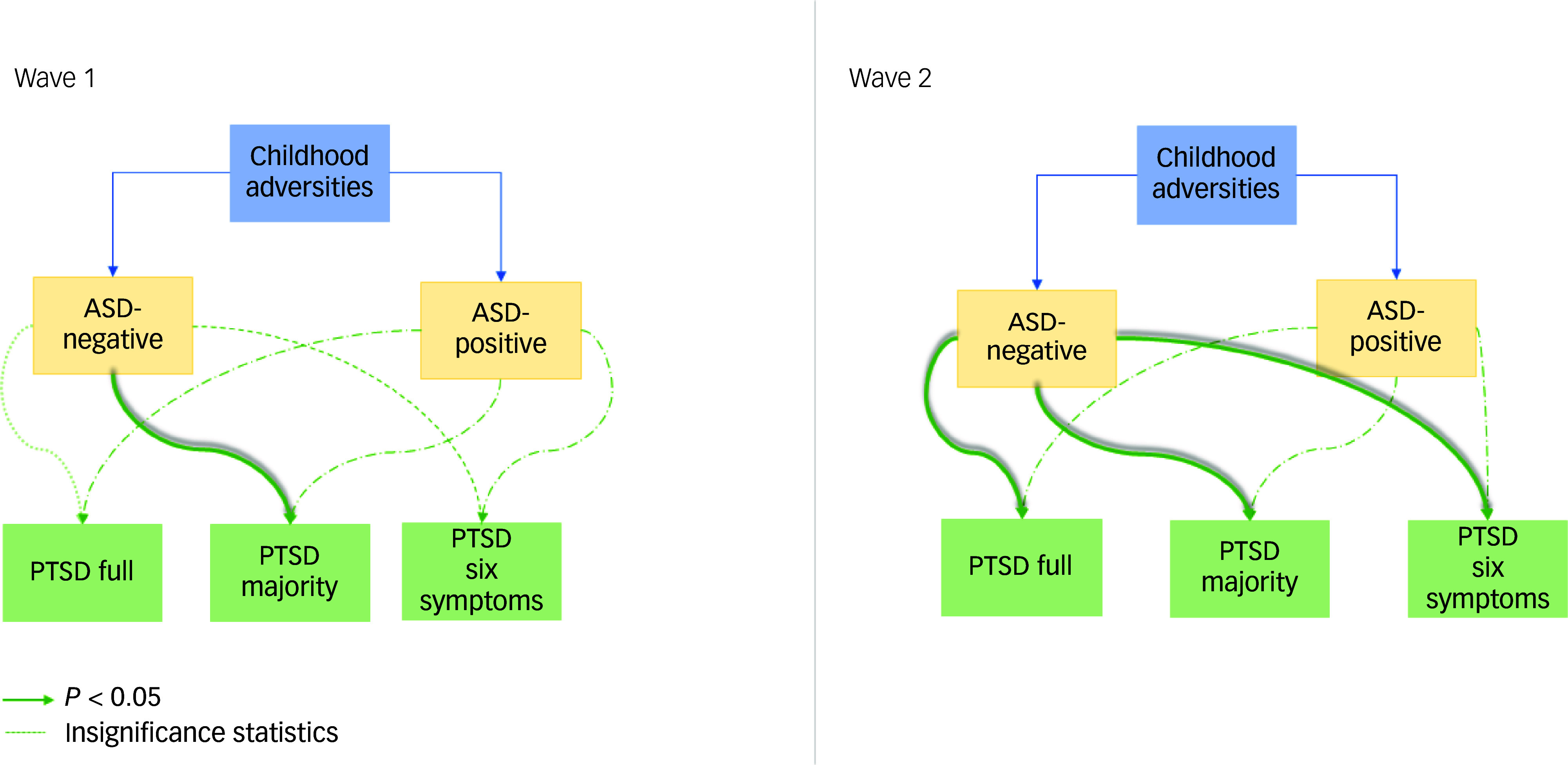
Predictive pathways from childhood adversity to post-traumatic stress disorder (PTSD) with and without acute stress disorder (ASD) mediation, based on multiple logistic regression models adjusted for age and gender (model 3, Tables 3 and 4).

To further illustrate these findings, Fig. 1 presents the predictive pathways from childhood adversity to PTSD, stratified by ASD status.

## Discussion

The introduction of ASD in the DSM-IV was intended to capture, early on, individuals at the highest risk of subsequent PTSD.^27^ Therefore, PTSD can be conceptualised as a continuation of ASD, suggesting that these two disorders are likely to share common predictors. Considering the well-documented association between childhood adversities and PTSD, it is reasonable to anticipate that these adversities may also serve as predictors of ASD.

### Childhood adversity and ASD

This was the case in our study, where sexual abuse emerged as a significant predictor of ASD at 9–15 days post-blast, whereas emotional abuse predicted ASD at 21–27 days post-blast. This finding is notable because, to our knowledge, the relationship between childhood adversities and ASD has not been thoroughly investigated previously. For instance, the retrospective matched-cohort study by Wang et al, which utilised the Taiwan National Health Insurance database to identify participants with a history of childhood abuse by using the ICD codes, measured the rates of multiple psychiatric disorders, including PTSD and ASD, but treated them as a combined entity (ASD/PTSD) rather than two distinct disorders.^19^ Nevertheless, childhood adversities were found to be strong predictors of ASD/PTSD, with an odds ratio of 3.27. Similarly, Maunder et al measured acute stress response to assess the link between childhood abuse/neglect, response to critical incidents and mental/physical symptoms in paramedics. Acute stress response, encompassing somatic, depressive and burnout symptoms, was measured over a 2-week period following critical incidents. Those with a history of childhood abuse/neglect showed heightened acute stress responses and higher depressive, physical and burnout scores.^20^ Although this study did not assess ASD according to diagnostic criteria (as ours did), it demonstrated that individuals with a history of childhood trauma experience heightened stress symptoms following adulthood incidents.

### Childhood adversity and proxy PTSD

As for the relationship between childhood adversities and proxy PTSD, our results pertaining to full threshold DSM-5 proxy PTSD are consistent with existing literature. However, no previous studies have, to our knowledge, examined the association between childhood adversity and subthreshold PTSD. This distinction is critical, as subthreshold diagnoses are associated with high distress, low quality of life and high comorbidities, yet are often overlooked in the full-threshold provisional diagnosis of PTSD.^28,29^ For instance, McLaughlin et al conducted a large-scale study involving 27 017 participants from the World Mental Health Surveys, identifying childhood adversities associated with PTSD. Among 12 adversities assessed, including interpersonal loss, parental maladjustment and maltreatment, individuals reporting physical abuse, sexual abuse, neglect and parental psychopathology were nearly twice as likely to develop PTSD after subsequent trauma (odds ratio 1.8), with sexual abuse and neglect emerging as the strongest predictors.^16^ In our present study, emotional abuse was also consistently associated with full threshold DSM-5 proxy PTSD and subthreshold proxy PTSD even after accounting for age and gender. Similarly, Widom’s study investigated the association between childhood abuse and neglect and subsequent PTSD, accounting for various familial, individual and lifestyle factors. Through matching victims of substantiated abuse and neglect with non-abused and non-neglected individuals followed into adulthood, the analysis revealed that childhood neglect was associated with an odds ratio of 1.75, indicating an increased susceptibility to PTSD.^30^

Interestingly, Sullivan et al conducted a study on 89 psychiatric in-patients, aiming to explore the impact of various subtypes of childhood on the severity of the individual PTSD symptom clusters (cluster B: re-experiencing, cluster C: avoidance and cluster E: arousal). The study found that sexual abuse predicted the re-experiencing symptom with an odds ratio of 1.24, whereas emotional abuse was a much stronger predictor of all the symptom cluster (re-experiencing: 1.43; avoidance and numbing: 1.62; arousal: 1.54), as well as overall PTSD severity (odds ratio 1.59).^31^

### Childhood adversity as a predictor of proxy PTSD: the intermediary role of ASD

Expanding on the observations that childhood adversities are predictive of both ASD and proxy PTSD, a pertinent question arises: Can childhood adversities directly predict proxy PTSD, bypassing ASD altogether?

When examining the longitudinal trajectory of childhood adversities in relation to ASD (within the first month post-blast) and proxy PTSD (6–7 months post-blast), we observed that participants without ASD in the month following the blast were more likely to develop subthreshold proxy PTSD (‘proxy PTSD majority’) if they reported at least one childhood adversity. This pattern was consistent at both time frames (9–15 days and 21–27 days post-blast).

As this is the first investigation, to our knowledge, into the longitudinal impact of childhood adversities on future proxy PTSD onset, both with and without going through ASD, a comparison with existing literature cannot be done. However, as this constitutes a novelty, it is imperative to try to explain the findings as to why individuals with a childhood history did not exhibit ASD early on, but later developed subthreshold proxy PTSD 6 months post-blast. Interestingly, among participants who developed ASD, a childhood history did not increase the risk of proxy PTSD 6 months later.

One potential explanation is that early exposure to adversities may foster post-traumatic growth, that is, positive psychological changes following exposure to trauma.^32^ Introduced by Tedeschi and Calhoun, post-traumatic growth refer to emotional and cognitive processing of the trauma that leads to positive personality changes and coping mechanisms,^33^ thereby enhancing resilience.^9^ This was evident in Cabrera’s study on deployed troops, where exposure to ACE was found to decrease reactivity in the face of subsequent trauma exposure, specifically combat exposure. Soldiers with a history of ACE exhibited lower scores indicating fewer stress symptoms following combat, compared with those with no such history of childhood adversity.^34^ Hence, it is plausible that some participants in our study developed resilience through post-traumatic growth, which protected them from ASD.

However, in the months following the blast, the situation in the country continued to deteriorate, with multiple collective stressors continuing to impact the participants, and this may have contributed to the onset of proxy PTSD. First of all, extensive media coverage displaying scenes of devastation may have retriggered memories of the blast, especially among healthcare workers at the most affected hospital in Beirut. They were simultaneously contending with infrastructure damage, a surge in COVID-19 cases and financial hardships following the economic crisis that began in 2020. The devaluation of the Lebanese currency (over 98%) and inflation exceeding 170% further amplified the stress.^35^

Such cumulative stressors may have contributed to the onset of proxy PTSD. Evidence suggests that repeated exposure to traumatic imagery, such as after the 9/11 attacks, leads to higher rates of PTSD among survivors.^36^ Similarly, the repeated exposure to distressing media can serve as an additional indirect re-exposure, thereby causing increased psychological arousal and retriggering trauma responses.^35^ Furthermore, working in healthcare during the pandemic puts individuals at greater risk of developing PTSD.^37^ Bryant’s 6-year longitudinal study on the trajectory of PTSD supports this. According to the sensitisation model of PTSD, individuals exposed to trauma may display exaggerated reactions to subsequent less severe stressors because of prior neural sensitisation.^38^ This proposed analogy provides a plausible explanation for why individuals who initially showed no signs of ASD later developed PTSD. The initial resilience derived from childhood adversities may have protected participants from ASD, but was eventually overcome by cumulative post-blast subsequent stressors, ultimately resulting in proxy PTSD.

Regardless of the mechanism, our findings represent progress in disaster mental health research. In the immediate aftermath of large-scale disasters (being man-made or natural), disaster response workers utilise the Psychological First Aid – Field Operations Guide, developed by the National Child Traumatic Stress Network and the National Center for PTSD, to assess survivors and provide early intervention.^39^ Our findings are particularly relevant in this context, emphasising the importance of assessing childhood adversities during such interviews, especially among survivors who did not exhibit ASD. The likelihood of these individuals developing proxy PTSD later, even if they initially showed no proxy PTSD symptoms, is significant. Therefore, early intervention is vital as a preventive measure.

### Strengths

This study offers novel insights with practical implications for improving public health responses in disaster settings. To our knowledge, it is the first study on the trajectory of childhood adversities leading to both full and subthreshold proxy PTSD, with and without the intermediary ASD diagnosis. Furthermore, it stands as the first robust study on the prediction of ASD diagnosis by childhood adversities. Furthermore, the study employed widely utilised assessment tools, which enhance the reliability and validity of the findings: the ASDS, which demonstrated strong internal consistency (Cronbach’s alpha of 0.94) in Karam et al’s study on the predictors of ASD;^40^ and the PCL-5, which is also among the most commonly used instruments for proxy DSM-5 PTSD diagnosis, and has an excellent internal consistency of 0.92 in this sample. Similarly, two validation studies on the ASDS and PCL-5 in a Lebanese population were published that included criterion validity^41^ and construct validity.^42^

### Limitations

This study has several limitations that warrant consideration. First, the relatively wide confidence intervals observed in some models may be partly explained by the small sample size in specific subgroups, which reduces the precision of the estimates and warrants cautious interpretation of the findings. Nevertheless, our response rates across 21–27 days and 6–7 months post-blast were 38 and 41.88% respectively, which is considered a high response rate in online-based surveys.^43^ Unfortunately, the response rate 9–15 days post-blast could not be obtained because of the emergency nature of the situation immediately following the blast and during polymerase chain reaction testing (the data collection was face to face). Future research could mitigate this by employing larger multi-site recruitment strategies to ensure sufficient power. Second, both ASD and proxy PTSD (full threshold and subthreshold) were assessed using screening tools rather than structured clinical interviews, potentially affecting the accuracy of the diagnoses. Although it is imperative to note that during times of disaster, conducting structured clinical interviews may not be feasible and many researchers, including those in our study, resort to using valid screening tools as a pragmatic approach to assess these disorders. We acknowledge that the prevalence estimates derived from self-reported questionnaires can differ substantially from those obtained via interviews. This underscores that our reported numbers, although based on a validated and comprehensive instrument, may still overestimate or underestimate true prevalence of both full and subthreshold proxy PTSD and ASD. Third, only three out of the eight childhood adversities documented in the literature were evaluated in this study, limiting the comprehensiveness of our assessment. Future studies may adopt more inclusive instruments, such as the full ACEs questionnaire, to capture a broader range of adverse experiences. Additionally, participants self-reported their history of childhood adversities, introducing the possibility of recall bias. This bias is a common occurrence when dealing with events from one’s past, as memory may not always be accurate or complete. However, given the nature of the study and the absence of alternative methods for collecting this data, relying on self-reports from adults about their childhood adversities was unavoidable. Moreover, the sample was self-selected rather than randomly selected, raising concerns about selection bias and generalisability of our results beyond our sample. To address this, researchers can consider random sampling. Additionally, this study does not have a baseline measurement for ASD and proxy PTSD, making it difficult to determine if some non-trauma specific symptoms predated the assessed trauma. Also, trauma exposure was assessed subjectively without severity measures. Lastly, another limitation pertains to the temporal overlap between the Beirut port blast and the COVID-19 pandemic, both of which represented major and concurrent stressors for healthcare workers. Although our instruments explicitly referred to the Beirut port blast as the index trauma when assessing acute and post-traumatic stress reactions, it is possible that participants’ responses were confounded by the broader psychosocial context of the pandemic. In such circumstances, distinguishing between distress specifically attributable to the blast and that stemming from ongoing pandemic-related challenges can be difficult. Examples of those non-specific distress symptoms are loss of interest in previously enjoyable activities, trouble falling asleep, being alert, feeling distant or cut off from people, and trouble experiencing positive feelings. Therefore, some of the reported symptoms may reflect a cumulative or interacting effect of both events or either one rather than a discrete trauma response to the blast alone.

### Future directions

For future research directions, longitudinal studies should be conducted across diverse populations, as our study focused solely on healthcare workers. Furthermore, to validate our theory regarding the development of PTSD without prior ASD, future studies should examine populations with minimal risk of continuous exposure to multiple stressors.

In conclusion, childhood adversities, specifically neglect, emerged as strong predictors of both ASD and proxy PTSD. The longitudinal analysis reveals the complex interplay between childhood adversities, ASD and subsequent proxy PTSD. Notably, healthcare workers who survived the Beirut port blast and who did not screen positive for ASD are at a significantly higher risk of developing subthreshold proxy PTSD later if they have a history of neglect during childhood. This underscores the critical importance of comprehensive assessments of childhood adversities in disaster response interventions and looking into subthreshold proxy PTSD diagnosis beyond the full threshold.

## Supporting information

Al Barathie et al. supplementary material Al Barathie et al. supplementary material

## Data Availability

The data are not publicly available due to restrictions, including information that could compromise the privacy of research participants.

## Supplementary material

The supplementary material is available online at https://doi.org/10.1192/bjo.2025.10948
