# Supplementary material for: Childhood adversities and post-traumatic stress: predictive pathways through acute stress disorder
Source: BJPsych Open. 2026 Jan 16;12(1):e40. doi: 10.1192/bjo.2025.10948 (PMC12835713; doi:10.1192/bjo.2025.10948)
Supplement: Al Barathie et al. supplementary material [file S2056472425109484sup001.docx]

Table S1. Distribution of participants across the study waves.

|  | Wave 1 | Wave 2 | Wave 3 |
| --- | --- | --- | --- |
| Time of assessment (post blast) | 9-15 days | 21-27 days | 6-7 months |
| Mode of assessment | Face to face: self-filled questionnaire in offices next to PCR screening area | Mass emails sent via MailChimp + SMS and WhatsApp reminders: self-filled questionnaire online | Mass emails sent via MailChimp + SMS and WhatsApp reminders + Personalized letters with QR codes: self-filled questionnaire using Chilean Platform |
| Total Number of participants | 570 | 733: 304 followed up from wave 1 | 808 out of which 426 have information in wave 3 and some information from either wave 1 and/or wave 2 (50 from wave 1 only; 209 from wave 2 only; 167 from waves 1 and 2) |
| Response Rate | NA* | 38% | 41.88% |

**In view of the urgency and the setting, we could not calculate the response rate because we failed to check how many refused to fill the survey, but refusals were estimated b our staff to be “low”.*
